# Calcium Extrusion Pump PMCA4: A New Player in Renal Calcium Handling?

**DOI:** 10.1371/journal.pone.0153483

**Published:** 2016-04-21

**Authors:** Ellen P. M. van Loon, Robert Little, Sukhpal Prehar, René J. M. Bindels, Elizabeth J. Cartwright, Joost G. J. Hoenderop

**Affiliations:** 1 Department of Physiology, Radboud Institute for Molecular Life Sciences, Radboud university medical center, Nijmegen, The Netherlands; 2 Institute of Cardiovascular Sciences, Manchester Academic Health Sciences Centre, University of Manchester, Manchester, United Kingdom; University Medical Center Utrecht, NETHERLANDS

## Abstract

Calcium (Ca^2+^) is vital for multiple processes in the body, and maintenance of the electrolyte concentration is required for everyday physiological function. In the kidney, and more specifically, in the late distal convoluted tubule and connecting tubule, the fine-tuning of Ca^2+^ reabsorption from the pro-urine takes place. Here, Ca^2+^ enters the epithelial cell via the transient receptor potential vanilloid receptor type 5 (TRPV5) channel, diffuses to the basolateral side bound to calbindin-D_28k_ and is extruded to the blood compartment via the Na^+^/Ca^2+^ exchanger 1 (NCX1) and the plasma membrane Ca^2+^ ATPase (PMCA). Traditionally, PMCA1 was considered to be the primary Ca^2+^ pump in this process. However, in recent studies TRPV5-expressing tubules were shown to highly express PMCA4. Therefore, PMCA4 may have a predominant role in renal Ca^2+^ handling. This study aimed to elucidate the role of PMCA4 in Ca^2+^ homeostasis by characterizing the Ca^2+^ balance, and renal and duodenal Ca^2+^-related gene expression in PMCA4 knockout mice. The daily water intake of PMCA4 knockout mice was significantly lower compared to wild type littermates. There was no significant difference in serum Ca^2+^ level or urinary Ca^2+^ excretion between groups. In addition, renal and duodenal mRNA expression levels of Ca^2+^-related genes, including TRPV5, TRPV6, calbindin-D_28k_, calbindin-D_9k_, NCX1 and PMCA1 were similar in wild type and knockout mice. Serum FGF23 levels were significantly increased in PMCA4 knockout mice. In conclusion, PMCA4 has no discernible role in normal renal Ca^2+^ handling as no urinary Ca^2+^ wasting was observed. Further investigation of the exact role of PMCA4 in the distal convoluted tubule and connecting tubule is required.

## Introduction

Calcium (Ca^2+^) is involved in several important processes in the body, including muscle contraction, bone mineralization and as a second messenger in multiple signal transduction pathways [1]. As a consequence, the plasma Ca^2+^ concentration is tightly controlled via absorption of dietary Ca^2+^ at the intestine, storage in bone and reabsorption by the kidney [2, 3]. In the kidney the majority of filtered Ca^2+^ is passively reabsorbed in the proximal part of the nephron, though fine-tuning occurs in the late distal convoluted tubule (DCT) and the connecting tubule (CNT) [4]. This is an active process where Ca^2+^ reabsorption can be regulated by hormones including parathyroid hormone (PTH) and active vitamin D (1,25(OH)_2_D_3_) [5–7]. In the late DCT and CNT Ca^2+^ enters the cell from the pro-urine via the apically expressed transient receptor potential vanilloid channel type 5 (TRPV5) [8, 9]. Subsequently, Ca^2+^ binds to calbindin-D_28k_ (CaBP_28k_) and/or calbindin-D_9k_ (CaBP_9k_) and this complex diffuses to the basolateral side, where Ca^2+^ is extruded via the Na^+^/Ca^2+^ exchanger (NCX1) or plasma membrane Ca^2+^ ATPase (PMCA) [10, 11].

PMCA is a member of the P-type ATPase family, and is related to the sarcoplasmatic/endoplasmatic reticulum Ca^2+^ ATPase pumps. Four different genes encode for PMCA 1 to 4. Both PMCA1 and 4 are ubiquitously expressed and have been suggested to have a housekeeping function, extruding Ca^2+^ from the cell [12]. PMCA1 knockout (KO) mice are embryonically lethal, whereas the PMCA4 KO mice are viable and appear healthy [13, 14]. This suggests that PMCA4 might have a more specific role than PMCA1. Indeed, PMCA4 has been shown to play a role in Ca^2+^ signaling in sperm motility, B-lymphocytes and cardiac nitric oxide signaling [13–16].

In the kidney, PMCA1 and 4 transcripts and protein are found in the proximal tubule, but higher expression has been shown at the distal part of the nephron [17, 18]. PMCA1 is considered as the predominant PMCA responsible for transcellular Ca^2+^ transport in the late DCT and CNT [18, 19]. Recently however, Alexander *et al*. investigated the exact localization of PMCA4 in the kidney, by co-staining the different tubular segments with representative markers [20]. They verified that PMCA4 was expressed highest in tubules that also expressed TRPV5. In addition, PMCA4 was decreased in TRPV5 KO mice, as were NCX1 and CaBP_28k_ [21, 22]. On the contrary, PMCA1 was not changed [22]. This suggests that PMCA4 may be the predominant PMCA form involved in distal transcellular Ca^2+^ reabsorption.

In this study we assessed the role of PMCA4 in renal Ca^2+^ handling. To this end, the PMCA4 KO mouse model was used and its Ca^2+^ balance was compared to wild type (WT) and heterozygous (HZ) littermates. There were no significant differences in serum Ca^2+^ levels or urinary Ca^2+^ excretion between groups or changes in expression of Ca^2+^-related genes.

## Material and Methods

### Ethics statement

This study was carried out in strict compliance with the United Kingdom Animals (Scientific Procedures) Act 1986. All experimental procedures were approved by the University of Manchester Ethics Committee (permit-no: 40/3625) and all efforts were made to minimize suffering of animals. A completed ARRIVE guidelines checklist is included in S1 Checklist.

### Animals

PMCA4 germline null mutant mice, as previously described [14], were maintained in a pathogen-free facility, housed under a 12 hour light/dark cycle, with *ad libitum* access to food and water. Experiments were performed with male WT (n = 10), HZ (n = 7) and KO (n = 10) mice when animals were aged 27–31 weeks old.

### Immunofluorescence

Immunofluorescence staining was used to detect PMCA4 protein expression in kidney sections, and was performed as described previously [23]. In short, PMCA4 staining was performed on 5 μm thick sections of 1% (w/v) periodate-lysine-paraformaldehyde-fixed mouse kidney samples. Sections were incubated overnight at 4°C with mouse anti-PMCA4 (1:100, ab2783, Abcam, Cambridge, UK) and incubated with Alexa Fluor 488 conjugated secondary antibody (1:300, Invitrogen, Carlsbad, CA, USA) for 2 hours. Images were collected with an AxioCam camera and AxioVision software (Zeiss, Sliedrecht, The Netherlands).

### Metabolic cage study

To collect urine, mice were housed individually in metabolic cages for 24 hours (from 10am to 10am) with *ad libitum* access to food and drinking water. The amount of water and food provided to each mouse was recorded at the start and after the 24-hour period, to determine amounts ingested. Following the metabolic cage housing, blood was collected via the jugular vein under isoflurane anesthesia and stored overnight at 4°C. All animals were humanely sacrificed by cervical dislocation. Kidneys were isolated, decapsulated, subdivided and snap frozen in liquid nitrogen. Duodenum segments were extracted, cleaned with PBS and snap frozen in liquid nitrogen. All samples were stored at -80°C until required. Serum was obtained following centrifugation of whole blood samples at 500*g* for 10 min, and frozen at -80°C until required. One KO mouse presented with fluid filled kidney cysts, and was not included in data analysis.

### Analytical procedures

Urinary osmolality was measured using an Advanced® Model 3320 Micro-Osmometer (Advanced instruments Inc., Norwood, MA, USA). A colorimetric assay was used to determine serum and urine Ca^2+^ concentrations as described previously [22]. Ca^2+^ measurements were verified using an internal control, which was a commercial serum standard (Precinorm U, Roche, Basel, Switzerland). Serum and urinary phosphate (P_i_) concentrations were determined by in-hospital services using automatic biochemical analyzers. Serum PTH levels were determined using the mouse PTH 1–84 ELISA kit (Immunotopics international, San Clemente, CA, USA) and serum Fibroblast Growth Factor (FGF)23 levels were determined with the mouse C-terminal FGF23 ELISA assay (Immunotopics International).

### Quantitative real-time PCR

Total RNA was isolated from kidneys and duodenum using TRIzol® reagent (Invitrogen) according to the manufacturer’s protocol and dissolved in 150 μl diethylpyrocarbonate (DEPC)-treated deionized water, and stored at -80°C. Thereafter, RNA was treated with RQ1 DNase (1U, Promega, Madison, WI, USA) and reverse transcription was performed using 1.5 μg RNA and Moloney murine leukemia virus reverse transcriptase (200 U, Invitrogen) for 1.5 hours following the manufacturer’s protocol, except that RNasin® (Promega) was used as an RNase inhibitor [22]. The obtained cDNA was used to determine mRNA levels of several Ca^2+^-related genes, as well as glyceraldehyde 3-phosphate dehydrogenase (GAPDH) as an endogenous control [22]. All the primers used (Biolegio, Nijmegen, The Netherlands) are listed in S1 Table. SYBR® Green mastermix (Bio-Rad, Veenendaal, The Netherlands) was used to perform quantitative real-time PCR according to manufacturer’s protocol and samples were measured on a CFX96 Bio-Rad analyzer (Bio-Rad). Gene expression was normalized for *Gapdh* expression and calculated according to the ΔΔCT method [24]. The PMCA4 real-time PCR product from WT, HZ and KO samples were run on a 2% (w/v) agarose gel electrophoresis.

### Protein isolation from kidney and immunoblotting

Total kidney protein lysates were prepared as described previously [25] and 30 μg protein was loaded to either 8% (w/v) or 12% (w/v) SDS-PAGE gel for NCX1 and CaBP_28k_, respectively and blotted to a PVDF membrane (Millipore, Billerica, MA, USA). Immunoblots were incubated overnight at 4°C either with mouse anti-CaBP_28k_ (1:5,000, clone CB-955, Sigma-Aldrich, Zwijndrecht, The Netherlands), mouse anti-NCX1 (1:500, ab6495, Abcam) or mouse anti-beta-actin (1:10,000, clone AC-15, Sigma-Aldrich). Immunoblots were enhanced using chemiluminescence (ECL, Pierce, Etten-Leur, The Netherlands) and analyzed using a ChemiDoc XRS (Bio-Rad) system. Semi-quantification was performed as described previously [26].

### Statistics

Values are expressed as mean ± S.E.M. Differences between the WT, HZ and PMCA4 KO mice were tested using a one-way ANOVA with Tukey post-hoc test. Differences were considered significantly different when P<0.05. Analysis of the dataset was performed using GraphPad Prism, version 6.0.

## Results

### Similar serum Ca^2+^ levels and urinary Ca^2+^ excretion in WT and PMCA4 KO mice

**Fig 1:**
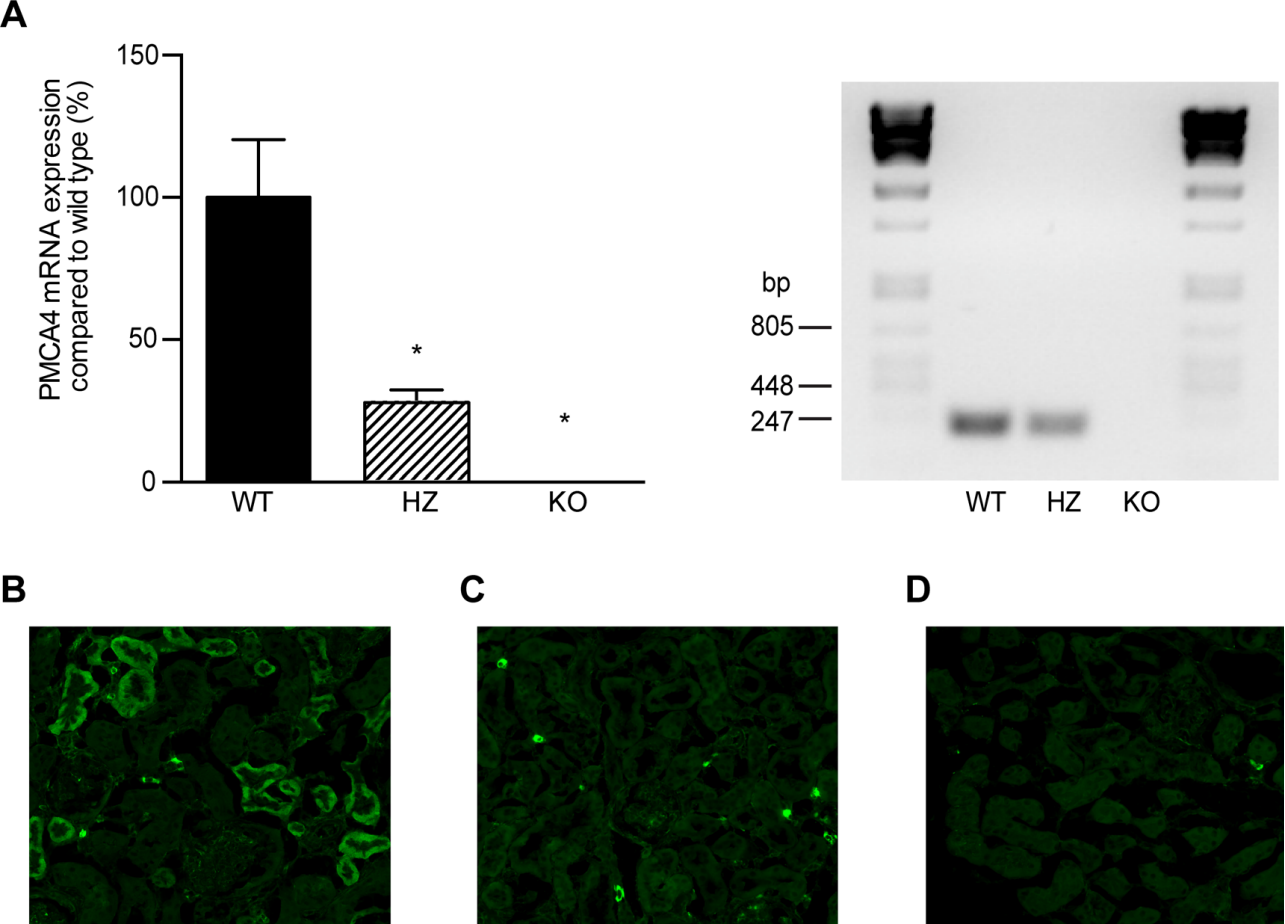
Confirmation of PMCA4 knockout in the kidney. (**A**) Renal relative mRNA expression levels (corrected for GAPDH) of PMCA4 in wild type (WT, n = 10), heterozygous (HZ, n = 7) and knockout (KO, n = 10) mice. Data represents mean ± S.E.M. *P<0.05 compared to WT. Real-time PCR products were subjected to 2% (w/v) agarose gel electrophoresis. (**B-D**) Representative immunofluorescence image for PMCA4 expression in mouse kidney cortex of wild type (**B**) and PMCA4 knockout mice (**C**). (**D**) Negative control without the primary antibody.

**Fig 2:**
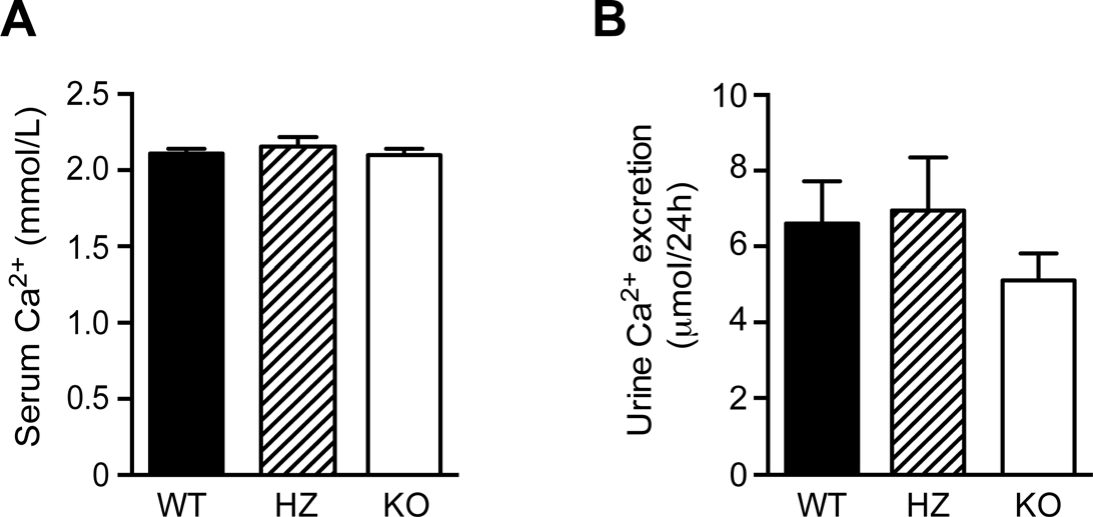
No difference in serum and urinary Ca^2+^ levels between the different PMCA4 genotypes. Serum Ca^2+^ (**A**) and 24-hour urinary Ca^2+^ excretion (**B**) in wild type (WT, n = 10), heterozygous (HZ, n = 7) and PMCA4 knockout (KO, n = 10) mice. Data represents mean ± S.E.M.

The role of PMCA4 in renal Ca^2+^ handling was investigated using PMCA4 WT, HZ and KO mice. By quantitative real-time PCR and immunofluorescence staining of the kidney cortex, PMCA4 mRNA and protein were not detected in kidneys from PMCA4 KO mice (Fig 1). Before the start of the animal experiment mice of the different groups were of similar body weight (data not shown). Subsequently, mice were housed in metabolic cages to collect 24-hour urine. There was no significant difference in body weight, kidney weight, food intake, diuresis or osmolality between the different groups (Table 1). Water intake was significantly decreased between WT and KO (Table 1). To examine if PMCA4 KO mice develop disturbances in their Ca^2+^ homeostasis, serum Ca^2+^ levels and 24-hour urinary Ca^2+^ excretion were measured; however, both were similar in all three groups (Fig 2).

**Table 1:** Characteristics of PMCA4 wild type, heterozygous and knockout mice.

	Wild type (n = 10)	Heterozygous (n = 7)	Knockout (n = 10)
**Body weight (g)**	27.0±0.4	28.7±0.7	28.3±0.9
**Kidney weight (g)**	0.36±0.01	0.38±0.01	0.37±0.01
**Food intake (g/24h)**	2.1±0.2	2.5±0.4	2.0±0.2
**Water intake (mL/24h)**	3.3±0.4	3.1±0.5	1.9 ±0.4*
**Diuresis (mL/24h)**	2.1±0.3	2.2±0.5	1.5±0.3
**Osmolality (mOsm/kg)**	1458±151	1644±299	2099±236

Wild type, heterozygous and knockout mice were housed for 24 hours in metabolic cages, after which body and kidney weight, food and water intake, diuresis and osmolality were measured. Data represents mean ± S.E.M.

*P<0.05 compared to wild type.

### No renal or duodenal compensation of Ca^2+^-related genes in PMCA4 KO mice

**Fig 3:**
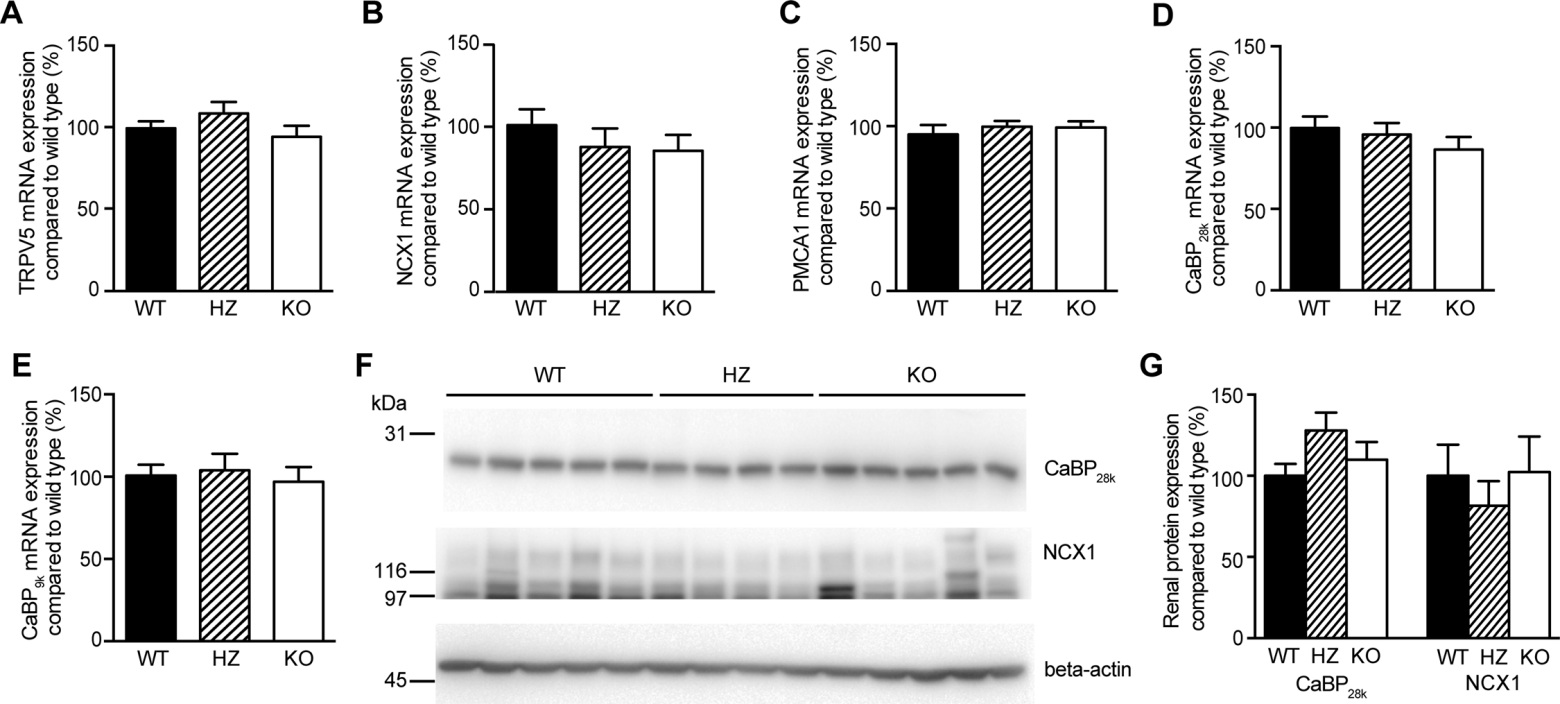
No effect of PMCA4 ablation on mRNA or protein levels of Ca^2+^-related genes in the kidney. Relative mRNA expression levels (corrected for GAPDH) of TRPV5 (**A**), NCX1 (**B**), PMCA1 (**C**), calbindin-D_28k_ (CaBP_28k_, **D**) and calbindin-D_9k_ (CaBP_9k_, **E**) were determined in kidneys of wild type (WT, n = 10), heterozygous (HZ, n = 7) and PMCA4 knockout mice (KO, n = 10). (**F**) Representative immunoblot of renal CaBP_28k_ and NCX1 protein expression in the three groups. Beta-actin was used as loading control. (**G**) Semi-quantification of immunoblots of CaBP_28k_ and NCX1, corrected for beta-actin and compared to WT. Data represents mean ± S.E.M.

**Fig 4:**
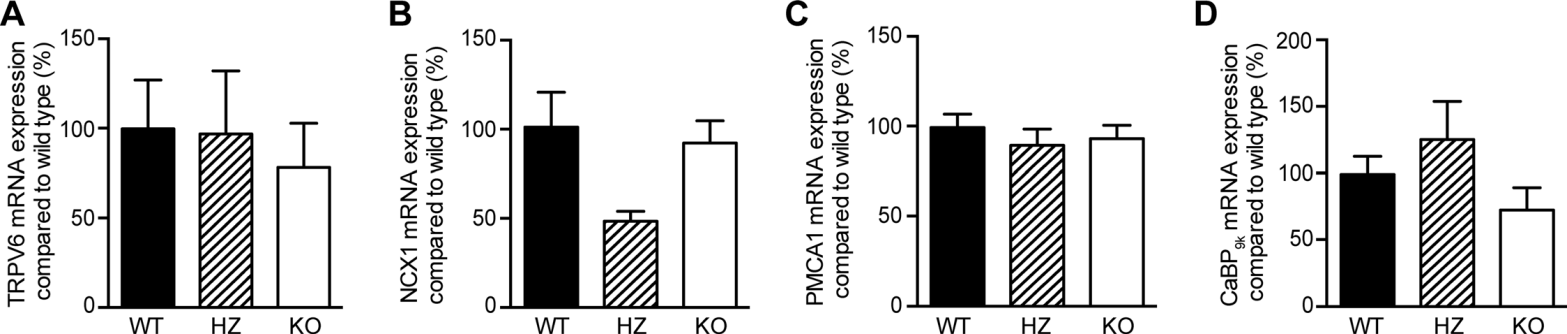
No effect of PMCA4 ablation on mRNA expression of Ca^2+^-related genes in duodenum. TRPV6 (**A**), NCX1 (**B**), PMCA1 (**C**) and calbindin-D_9k_ (CaBP_9k_, **D**) relative mRNA expression levels (corrected for GAPDH) were analyzed in the duodenum of wild type (WT, n = 10), heterozygous (HZ, n = 7) and PMCA4 knockout mice (KO, n = 10). Data represents mean ± S.E.M.

Renal mRNA expression of Ca^2+^-related genes was analyzed to determine possible compensation mechanisms following PMCA4 ablation. Expression of the epithelial Ca^2+^ channel TRPV5, the so-called Ca^2+^ gatekeeper of the late DCT and CNT [27], was not significantly different between groups (Fig 3A). In addition, other genes involved in Ca^2+^ reabsorption in the late DCT and CNT, including NCX1, PMCA1, CaBP_28k_ and CaBP_9k_, were similar between the genotypes (Fig 3B–3E). In addition, renal protein expression of CaBP_28k_ and NCX1 was not significantly different between groups (Fig 3F and 3G). Besides kidney, duodenal mRNA expression of Ca^2+^-related genes was measured to establish if potential compensation occurred in the duodenum. No difference was found for TRPV6, the active Ca^2+^ absorption channel in the duodenum [28] (Fig 4A). Moreover, NCX1, PMCA1 and CaBP_9k_ were comparable between the three genotypes (Fig 4B–4D).

### PMCA4 KO mice show increased FGF23 levels

**Fig 5:**
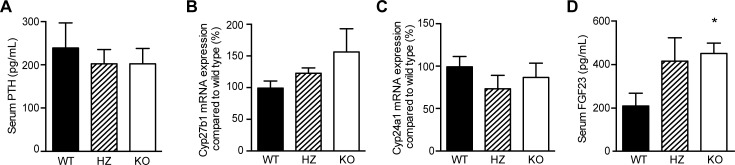
**PMCA4 ablation does not affect serum PTH but increases FGF23 serum levels** (**A**) Serum PTH levels were measured in wild type (WT, n = 10), heterozygous (HZ, n = 7) and PMCA4 knockout mice (KO, n = 10). Relative mRNA expression levels (corrected for GAPDH) of the renal vitamin D activating enzyme Cyp27b1 (**B**) and vitamin D degrading enzyme Cyp24a1 (**C**). (**D**) Serum FGF23 levels were significantly higher in KO mice compared to WT. Data represents mean ± S.E.M, *P<0.05 compared to WT.

In order to investigate whether hormonal Ca^2+^ regulation is changed in PMCA4 KO mice, PTH was measured in serum. No significant difference was present between the three groups of mice (Fig 5A). Moreover, renal mRNA expression of vitamin D activating (Cyp27b1) and breakdown (Cyp24a1) enzymes was investigated, and their expression was found not to significantly differ between genotypes (Fig 5B and 5C). Serum FGF23 levels were, however, significantly higher in PMCA4 KO mice compared to WT (Fig 4D). Since FGF23 is involved in regulating P_i_ homeostasis, serum P_i_ and 24-hour urinary P_i_ excretion were determined. There were, however, no significant differences between groups (S1 Fig). In addition, renal expression of klotho and Na^+^/P_i_ co-transporters NaPi-IIa and NaPi-IIc were determined, though also here no significant differences were observed (S1 Fig).

## Discussion

Importantly, for the first time, this study aimed to characterize the renal role of PMCA4. PMCA4 has recently been localized in Ca^2+^ transporting epithelial cells, promoting speculation about its functional role in transcellular Ca^2+^ transport at the distal nephron [20, 22]. In this study we show that ablation of PMCA4 has no significant effect on serum Ca^2+^ level or on renal Ca^2+^ excretion. Moreover, PMCA4 KO mice did not show Ca^2+^-related mRNA compensation in the kidney or duodenum.

The Ca^2+^ concentration in the body needs to be tightly regulated for multiple physiological processes [3, 29]. Disturbances in Ca^2+^ homeostasis can result in osteoporosis, heart failure or kidney stones [30, 31]. The late DCT and CNT are responsible for regulated active Ca^2+^ uptake, via the transcellular pathway. On the apical side, Ca^2+^ enters the cell via TRPV5, whereas NCX1 and PMCA1 and/or 4, located basolaterally, are responsible for Ca^2+^ extrusion [10,11, 22]. It was considered that PMCA1 was responsible for late DCT and CNT Ca^2+^ extrusion, since in the duodenum it is the principal Ca^2+^ efflux mechanism [19, 32]. However, Van der Hagen *et al*. showed that there was relatively more mRNA expression of PMCA4 in the late DCT and CNT compared to total kidney, whereas PMCA1 was not enriched in this segment [22]. This was confirmed at the protein level, where the most intense expression of PMCA4 in the kidney was observed in TRPV5 expressing tubules [20, 22]. In addition, the TRPV5 KO mouse showed a decrease in mRNA levels of CaBP_28k,_ NCX1 and PMCA4, but not PMCA1 [22]. Furthermore, parathyroidectomized rats, klotho KO mice and 1α-hydroxylase KO mice showed co-regulation of TRPV5, CaBP_28k_ and NCX1, but not of PMCA1 [5, 6, 33]. Therefore, we hypothesized that PMCA4, rather than PMCA1, is the predominant pump for renal Ca^2+^ homeostasis in the late DCT and CNT. However, in this study the PMCA4 KO mice did not show an increase in urinary Ca^2+^ excretion. PMCA1 and/or NCX1 might have compensated for the ablation of PMCA4, normalizing serum Ca^2+^ and urinary Ca^2+^ excretion. However, there was no significant upregulation of NCX1 at gene and protein level, or increased mRNA expression for PMCA1 in PMCA4 KO mice. Total ablation of either NCX1 or PMCA1 is embryonic lethal in mice, hence their role in Ca^2+^ extrusion in late DCT and CNT cannot be determined by using a global KO [13, 34]. On the contrary, TRPV5 KO mice are viable but present with severe hypercalciuria [21, 35]. Mice with depletion of the Ca^2+^ shuttling protein CaBP_28k_ show varying results concerning their Ca^2+^ excretion, with no difference or increased Ca^2+^ excretion compared to WT mice, depending on the study [27, 36–38]. In addition, Gkika *et al*. treated CaBP_28k_ KO mice with a low and high Ca^2+^ diet (0.02% w/w or 2% w/w), and did not observe a disturbance in Ca^2+^ homeostasis, even though CaBP_28k_ is involved in transcellular Ca^2+^ transport [27]. This suggests that we cannot necessarily rule out PMCA4 having a role in renal Ca^2+^ handling even though the PMCA4 KO mouse does not show a changed Ca^2+^ homeostasis.

At the intestinal level the PMCA4 KO mice did not show a difference in the Ca^2+^-regulating genes, indicating that no compensation occurred. Recently Alexander *et al*., have shown that PMCA4 is located in the smooth muscle layer of the intestine, and not in the enterocytes, where PMCA1 was more highly detected [20], suggesting that PMCA1, rather than PMCA4, is primarily involved in transcellular Ca^2+^ transport in the duodenum [20, 28, 39]. An earlier study has shown that PMCA4 KO mice presented with lower trabecular bone volume, lower bone mineral density and an increase in osteoclast surface area compared to WT mice [40]. Bone could be a source of Ca^2+^ to maintain a normal serum Ca^2+^ level; however, there was no indication of renal Ca^2+^ wasting in our mice studied here.

Ca^2+^ homeostasis is mainly regulated by PTH and 1,25(OH)_2_D_3_ in response to low serum Ca^2+^ levels [41]. The PMCA4 KO mice do not show differences in Cyp27b1 or Cyp24a1 mRNA, nor in serum PTH, compared to WT mice. This is consistent with our other observations where PTH- and 1,25(OH)_2_D_3_-regulated genes, such as TRPV5 and TRPV6, are not changed [5, 6, 42]. Moreover, PMCA4 KO mice presented with significantly lower water intake. However, since there were no changes in body weight or urinary excretion, this could have been caused by biological variation. In addition, we know of no reports indicating an involvement of PMCA4 in thirst and/or water homeostasis. Interestingly, FGF23 levels were increased in PMCA4 KO mice. In the kidney, FGF23 decreases NaPi-IIa and NaPi-IIc expression, resulting in increased urinary phosphate excretion [43]. In addition, it downregulates Cyp27b1 and increases Cyp24a1 expression [44]. These consequences were not observed in PMCA4 KO mice. This suggests that PMCA4 might be involved in FGF23 production and/or signaling, although more research is necessary to confirm this. Besides its role as general Ca^2+^ extruder, others have already shown the involvement of PMCA4 in signaling transducing pathways as well [45, 46].

In conclusion, PMCA4 does not seem to be vital for basal renal Ca^2+^ handling. However, the presence of PMCA4 in specific segments of the kidney points to the molecule having a different or additional role compared to PMCA1 and therefore, their exact roles should be investigated in more detail.

## Supporting Information

S1 Checklist The ARRIVE checklist. (PDF)

S1 Dataset Metabolic cage data. (XLSX)

S1 Fig No difference in serum P_i_ or 24-hour urinary P_i_ excretion between the different genotypes. Serum P_i_ (**A**) and 24-hour urinary P_i_ excretion (**B**) in wild type (WT, n = 10), heterozygous (HZ, n = 7) and knockout (KO, n = 10) mice. Relative mRNA expression of NaPi-IIa (**C**), NaPi-IIc (**D**) and klotho (**E**) were determined in the kidney. Data represents mean ± S.E.M.
(TIF)

S1 Table Primer sequences used for real-time PCR. (DOCX)
