# Supplementary figures and images for: Calcium Extrusion Pump PMCA4: A New Player in Renal Calcium Handling?
Source: PLoS One. 2016 Apr 21;11(4):e0153483. doi: 10.1371/journal.pone.0153483 (PMC4839660; doi:10.1371/journal.pone.0153483)

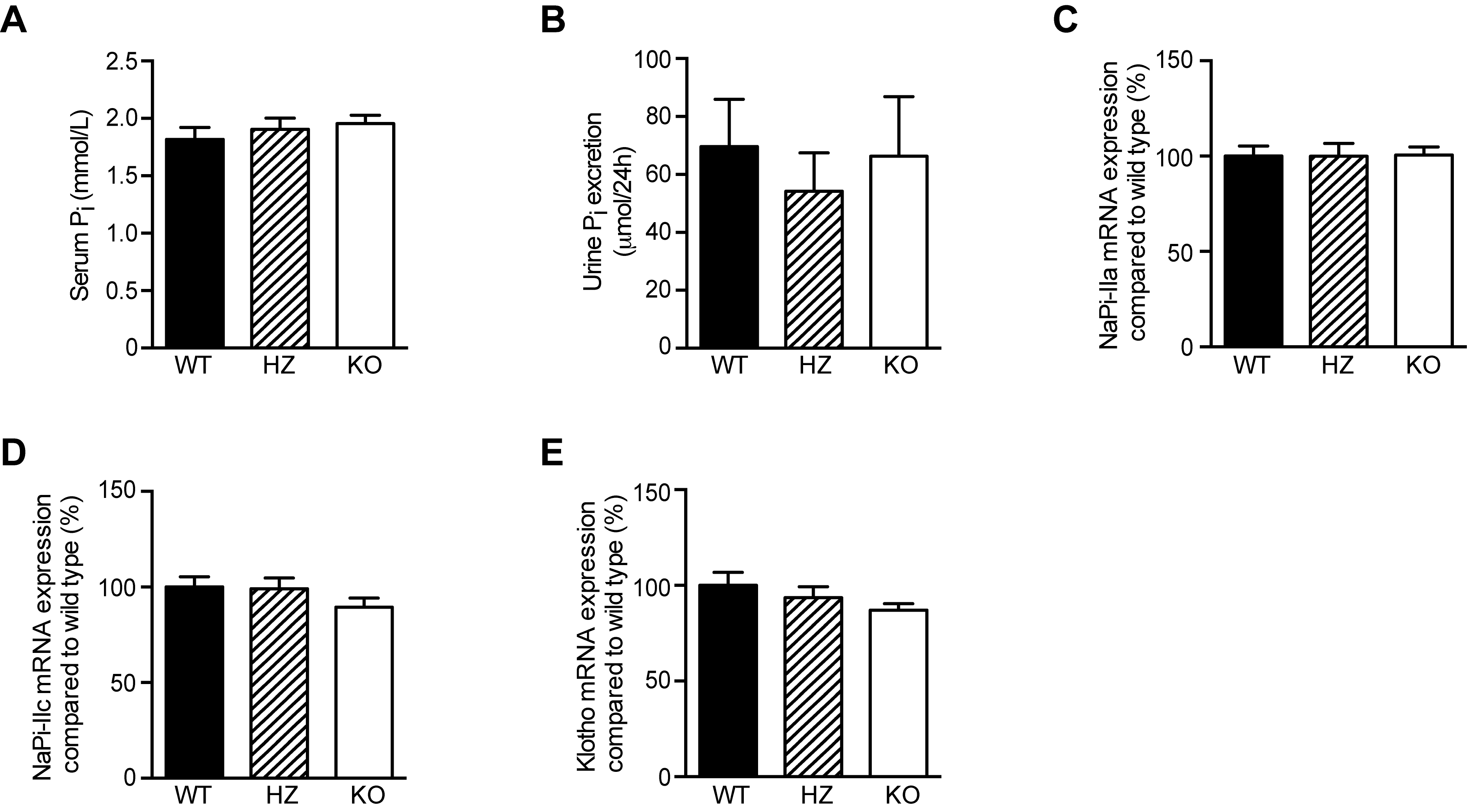

Supplement: S1 Fig — Serum Pi (A) and 24-hour urinary Pi excretion (B) in wild type (WT, n = 10), heterozygous (HZ, n = 7) and knockout (KO, n = 10) mice. Relative mRNA expression of NaPi-IIa (C), NaPi-IIc (D) and klotho (E) were determined in the kidney. Data represents mean ± S.E.M. (TIF) [file pone.0153483.s003.tif]
