# Supplementary material for: Calcium Extrusion Pump PMCA4: A New Player in Renal Calcium Handling?
Source: PLoS One. 2016 Apr 21;11(4):e0153483. doi: 10.1371/journal.pone.0153483 (PMC4839660; doi:10.1371/journal.pone.0153483)
Supplement: S1 Table — (DOCX) [file pone.0153483.s004.docx]

**S1 Table. Primer sequences used for real-time PCR**

| **Gene** | **Forward primer 5’ – 3’** | **Reverse primer 5’ – 3’** |
| --- | --- | --- |
| **GAPDH** | TAACATCAAATGGGGTGAGG | GGTTCACACCCATCACAAAC |
| **TRPV5** | CTGGAGCTTGTGGTTTCCTC | TCCACTTCAGGCTCACCAG |
| **TRPV6** | GGCCTCACAACCTCATTTAC | CTCAATGAGCAGTCTAACAATC |
| **CaBP_9K_** | GATGGGAAGCTGGAATTAACTGA | CCACACATTTTGATTCCCTGAA |
| **CaBP_28k_** | GACGGAAGTGGTTACCTGGA | ATTTCCGGTGATAGCTCCAA |
| **NCX1** | GTGACTGCCGTTGTGTTTGT | GCATACTGGTCCTGGGTAGC |
| **PMCA1** | GTCACCGGCCTTACGTGTAT | TCCAGCCCTCTGACATTTCT |
| **PMCA4** | CTTAATGGACCTGCGAAAGC | ATCTGCAGGGTTCCCAGATA |
| **Cyp27b1** | GTGTTGAGATTGTACCCTGTGG | TGGGGAATTACATAGTTTCCTACAC |
| **Cyp24a1** | GGAGTCCATGAGGCTTACCC | GGTAGCGTGTATTCACCCAGA |
| **NaPi-IIa** | TCAGGAAGAGGAGCAAAAGC | AAAGGAAAGCCAGCATCAGA |
| **NaPi-IIc** | GTGGTCAGCAGCTTTCTCAA | ACAGCACCACATTGTCCTTG |
| **Klotho** | GGTTGCCCACAACCTACTTT | TGGGAGCTTAAGGCGATAGA |

The forward and reverse mouse primers of the several genes used in quantitative real-time PCR of kidney and duodenal mRNA. GAPDH (glyceraldehyde 3-phosphate dehydrogenase); TRPV5 and 6 (transient receptor potential vanilloid member 5 and 6); CaBP_9K_ and CaBP_28K_ (calbindin-D_9k_ and calbindin-D_28k_); NCX1 (Na^+^/Ca^2+^ exchanger 1); PMCA1 and 4 (plasma membrane Ca^2+^ ATPase 1 and 4); Cyp27b1 (cytochrome p450 27b1); Cyp24a1 (cytochrome p450 24a1), NaPi-IIa and c (type II Na^+^/Pi co-transporter a and c.
